# Prevalence of Vitamin B12 deficiency in patients of type 2 diabetes mellitus on metformin: A case control study from Pakistan

**DOI:** 10.11604/pamj.2013.16.67.2800

**Published:** 2013-10-25

**Authors:** Raheel Iftikhar, Sultan Mehmood Kamran, Adnan Qadir, Zohaib Iqbal, Hassan bin Usman

**Affiliations:** 1 Combined Military hospital kharian, Pakistan

**Keywords:** Diabetes Mellitus, Vitamin B12 deiciency, Metformin

## Abstract

**Introduction:**

Diabetes Mellitus is the most common endocrine disorder and metformin is the most commonly prescribed oral hypoglycemic agent. Metformin is well known to cause viamin B12 deficiency due to effect on calcium-dependent membrane action in the terminal ileum leading to malabsorption of vitamin B12. The purpose of this study is to determine prevalence and associations of Vitamin B12 deficiency in patients of type 2 diabetes mellitus treated with metformin.

**Methods:**

This case control study was carried out in department of medicine, Combined Military Hospital, Kharian from 1^st^ Jan 2012 to 30 december 2012. We enrolled 114 outdoor patients of type 2 diabetes mellitus currently on metformin for atleast 12 months, by consecutive sampling, and 105 age and sex matched patients taken as control. Patients with vitamin B12 levels of less than 150 pg/ml were said to be B12 deficient. The results were analyzed on SPSS version 16.

**Results:**

Serum B12 levels were low in 35 patients (31%) on metformin as compared to only 9 patients (8.6%) among controls,(p value 0.002). Mean B12 levels were significantly low in metformin group 311 pg/ml (±194.4), p value 0.03. Dose of metformin had inverse correlation with B12 levels and the difference was statistically significant with p-value < 0.001.

**Conclusion:**

Our study demonstrated significantly high prevalence of vitamin B12 deficiency in patients treated with metformin with significant effect of dose and duration of metformin use on B12 levels. Physicians must recognize this important fact and screen diabetics on metformin therapy for underlying B12 deficiency.

## Introduction

Diabetes mellitus was reported to be the sixth leading cause of death listed on US death certificates in 2010 [1]. Ramachandran and Colleagues in 2012 documented that Prevelance of diabetes mellitus in Pakistan is 7.7% in rural and 10.6% in urban population with more than 7.2 million people suffering from this illness [2]. Metformin is most commonly prescribed oral hypoglycemic in patients with type 2 diabetes mellitus [3]. Because it is relatively well tolerated in most of the patients except for mild gastrointestinal side effects, most of the patients continue to take it for years. One of the documented side effects of metformin is vitamin B12 deficiency but it is almost always overlooked and seldom investigated [3]. Many of patients on metformin develop Vitamin B12 deficiency, consequently developing paraesthesias and anemia which is falsely attribute to underlying Diabetes mellitus by physicians and so never addressed [4]. As per literature search in Pubmed and Pakmedinet using keywords vitamin B12 and metformin, no studies on Pakistani diabetic Population were found till January 2013.

This study is designed to determine prevalence of vitamin B12 deficiency in patients on metformin and to evaluate factors associated with vitamin B12 deficiency in patints on metformin.

## Methods

This case control study was carried out at Outpatient Department of Medicine, Combined Military Hospital, Kharian in 01 year from 1^st^ Jan 2012 to 30^th^ December 2012. We selected 114 patients of type 2 diabetes mellitus on metformin for atleast 12 months. Control group comprised of 105 patients of type 2 diabetes mellitus with no history of metformin use in last 2 years. Sample size was calculated using Raosoft sample size calculator taking 95% confidence level, 5% margin of error, and response distribution of 50%. Both cases and controls were age and sex matched. Patients included in study were previously diagnosed outpatients of Type 2 diabetes mellitus between 40 to 70 years. Patients of both genders were included. Patients were excluded from study if they had history of anemia, prior transfusion, thyroid illness, alcohol intake, renal insufficiency, prior gastric surgery, patients on current parenteral or enteral nutritional support , those with malabsoprtion syndrome, use of B12 supplements (oral or parenteral), vegetarians, proton pump inhibitors. Informed written consent was taken from all participants and approval from ethical committee combined military hospital kharian was sought.

Patients to be tested were asked to come to laboratory, sit on a chair, roll up their sleeves above elbow. Venous blood samples were collected using full aseptic measures. Blood samples were kept at -30 degree celsius, kept in closed bottles which were held in vertical position. Samples were analysed the same day for B12 levels using DXI automated analyser. Glycosylated hemoglobin (HbA1c) measurement was done using DXC-600 automated analyser. The results were verified by pathologist. Data collected for each patient included age, gender, hemoglobin, duration of diabetes mellitus, dose of metformin, duration of metformin use. All data was entered in a predesigned Performa.

All the data was entered in computer software Statistical Package for Social Sciences (version 16.0). Descriptive statistics were applied out to summarize the data. Mean and standard deviation (±SD) was calculated for all the quantitative variables i.e. age, duration of metformin use, dose of metformin, serum vitamin b12 levels, glycosylated hemoglobin. Frequency and percentages were calculated for qualitative variables i.e. gender, vitamin b12 deficiency. Data was analysed using students t-test and X^2^ analysis. Mann-Whitney U test was applied for data which was skewed. Correlation between variables was also analysed using bivariate analysis with pearson equation wherever required. P value of less than 0.05 was considered statistically significant.

## Results

Total of 114 patients with type 2 diabetes mellitus on metformin (67 males, 47 females) were selected for study and 105 patients with type 2 diabetes mellitus not on metformin were taken as control ( 61 males, 44 females). Controls were matched with cases for age, gender, duration of diabetes mellitus and glycosylated Hemoglobin ( HbA1c).

### Sociodemographic characteristics

Mean age among metfromin group was 56 ± 8.02 years while it was 55± 9.02 years among control group. Mean duration of diabetes was 8.96± 4.74 years among patients on metformin while it was 8.82 ±4.72 years among control group. Mean glycosylated hemoglobin levels were 8.35% ± .87 among cases and 8.32%± .88 among controls. No significant association for age, gender, duration of diabetes mellitus, HbA1c, MCV was found between patients on metformin and control group. There was a trend towards relatively high MCV in patients on metformin (92 fl ± 4.57) as compared to controls (88 fl ± 2.52), the difference was not statistically significant (Table 1).

**Table 1:** Demographic characteristics of study group

Demographics	Patients on Metformin	Control group	P value
**Age (years) mean (SD)**	56 (± 8.02)	55 (± 9.02)	0.57
**Male n (%))**	67 (58%)	61(58%)	0.43
**Females (n(%))**	47(42%)	44(42%)	0.19
**Duration of diabetes (yrs) (mean(SD))**	8.96 (± 4.742)	8.82(± 4.721)	0.78
**Glycosylated hemoglobin (%) (mean(SD))**	8.35% (± .87)	8.32 (±.88)	0.46
**MCV**	92 (± 4.57)	88 (2.52)	0.32
**Vitamin B12 levels pg/ml(mean(SD))**	311 (±194.4)	414(± 223.5)	0.03
**Vitamin B12 deficiency (n(%))**	35 (31%)	9 (8.6%)	0.002

### Clinical Characteristics

**Figure 1:**
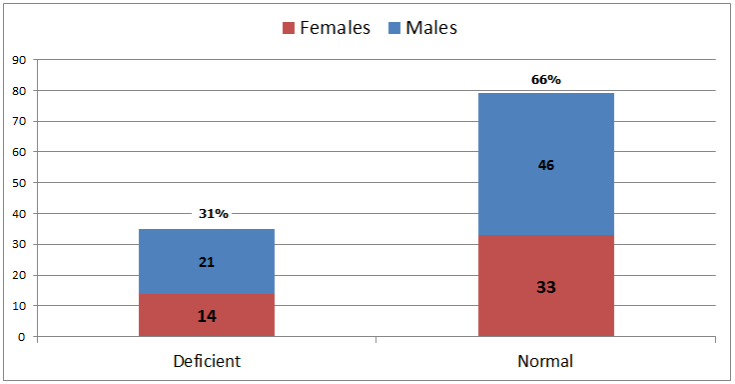
Prevelance of B12 deficiency in patients on metformin

There was a significant correlation of mean B12 levels and prevalence of B12 deficiency between both study groups. In patients on metformin, prevalence of vitamin B12 deficiency was 31%, (35 out of 114) ,while in control group prevalence of vitamin B12 deficiency was only 8.6% ( 9 patients) (Figure 1). Serum B12 levels among cases ranged from 57 pg/ml to 980 pg/ml with mean B12 levels of 311 pg/ml (± 194.4) while mean B12 levels among control was 414 pg/ml (± 223.5).

Both the duration and dose of metformin was found to have stastically significant affect on B12 levels (p value < 0.05). Mean B12 levels in patients on metformin for less than 24 months were 414 pg/ml (± 202) while those on more than 24 months was 188 pg/ml (± 78) with p-value <0.002. When comparing patients on metformin for less than 24 months with those on metfromin for more than 24 months in B12 deficient patients results were stastically significant with p-value< 0.001 (Table 2).

**Table 2:** Demographic characteristics of patient on metformin

Demographics	Vitamin B12 levels		P value
	Deficient ≤ 150 pg/ml	Normal >150 pg/ml	
**Number of patients N(%)**	35 (30.7%)	79(69.3%)	0.57
**Males n (%)**	21(31.3%)	46(68.7%)	0.18
**Females n(%)**	14(29.7%)	33(70.3%)	0.25
**Age years (mean, SD)**	55.97(±7.94)	55.99(±8.11)	0.992
**Vitamin B12 levels (mean, SD)**	131(±42.66)	391(±181.49)	.00001
**Dose of metformin mg (mean, SD)**	2042 (±560.61)	1607(±648.82)	.0003
**MCV**	98.5 (±6.84)	91(±5.98)	0.04
**Duration of metformin use months (mean, SD)**	28.2(±9.8)	18.8(±10.2)	0.001
**Duration of Diabetes years (mean, SD)**	9.89(±4.46)	8.54(±4.83)	0.082
**Glycosylated Hemoglobin HbA1c (%) SD**	8.68(±.90)	8.33(±.87)	0.401

### Effect of metformin dose

**Figure 2:**
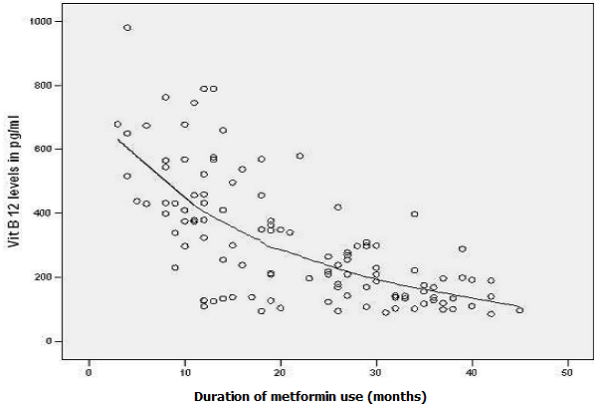
Inverse relation between duration of metformin use and vitamin B12 levels

We also correlated effects of dose of metformin on B12 deficinecy among patients taking less than 1500 mg and more than 1500 mg. Mean dose among B12 deficient patient was 2100 mg (± 497) while in patients with normal B12 levels mean dose was 1582mg(± 632). The dose of metformin had inverse correlation with B12 levels and the difference was statistically significant with p-value < 0.001 (Figure 2). There was no significant association between gender and Vitamin B12 levels among cases and controls. Correlation between HbA1c and b12 deficiency was also insignificant statistically with p-value 0.401.

## Discussion

Majority of patients with type 2 diabetes are prescribed metformin. One of documented side effect of metformin is vitamin B12 deficiency [3, 5]. The B12-intrinsic factor complex uptake by ileal cell membrane receptors is known to be calcium-dependent, and metformin affects calcium dependent membrane action, resulting in B12 deficiency [6]. Only limited data is available on this topic As per literature search no study has ever been done on Pakistani populution to determine prevalence of vitamin B12 deficiency in diabetic patients on metformin. In our study we found significantly lower B12 levels in patients on metformin as compared to controls with 31 percent patients on metformin found to be vitamin B12 deficient, comparing to only 9 percent deficiency among controls. These results are similar to a study performed in 2011 in irish population by Marar.O and colleagues [7]. They took informed consent and collected data on predesigned performa. Exclusion criteria was similar to our study but minimum duration of metformin use for inclusion in study was 18 months as compared to 12 months in our study. Moreover demographic data collected by them also included hemoglobin, folate, TSH, serum creatinine, presence of neuropathy. They evaluated association of metformin use and B12 deficiency with anemia, neuropathy. However mean age of patients were 7-8 years higher in their study. Additionally Marar.O and colleagues defined vitamin B12 deficiency consisting of three subdivisions as mild, moderate, severe . Therefore, it can be said that Marar.O and colleagues used more comprehensive and refined analysis in their study group. Previous study in 2009 by, Pflipson et al, on diabetic population has revealed B12 deficiency in range of 22 percent but the study population was evaluated using past medical records, survey for use of insulin, other hypoglycemic agents [8]. They took levels of less than 100 pg/ml as vitamin B12 deficiency. Moreover methylmalonic acid levels were also measured in this study, thus improving sensitivity. Similarly Ting et al, showed duration and dose of metformin as high risk factors for developing Vitamin B12 deficiency [9]. These results were very similar to our study showing significant association and inverse relation of duration and dose of metformin with B12 levels. A similar study performed in America didn't find any significant corelation between metformin use and B12 deficiency, however there was a trend towards lower B12 levels in patients on long term metformin [10].

A similar study performed in Brazil in 2010 showed low B12 levels (<125 pg/ml) in 6.9% and possibly low (125-250 pg/ml) in 36.8% of patients, results comparable to our study [11]. In contrast to previous studies [7–8, 12], we found a significant correlation between MCV among patients on metformin having B12 deficiency as compared to those who are not deficient.

Many patients with B12 (Cbl) deficiency have no or only mild anemia, and macrocytosis may be masked by a concurrent disorder (eg, iron deficiency, thalassemia) [13]. In one series, for example, the diagnosis of Cbl deficiency was confirmed in patients in whom only 29 percent had anemia, and only 36 percent had an MCV >100 fL [14]. Thus it is prudent to screen diabetics on metformin for underlying B12 deficiency. The value of routine screening for B12 deficiency (recommended by some) is unknown, but the clinician must be aware of this association. Bivariate analysis of our study demonstrated there was no significant association between age, gender, HbA1C and B12 deficiency.

One of limitation of our study was that we didn't measured methylmalonic acid levels which can improve the sensitivity of results by identifying B12 deficiency in early asymptomatic stage [15]. Serum concentrations of homocysteine (HC) as well as serum and urinary concentrations of methylmalonic acid (MMA) are elevated in Cbl deficiency, due to a decreased rate of metabolism [16]. In comparison, only HC is elevated in folate deficiency, since folate does not participate in MMA metabolism [17]. Previous study by, Pflipson et al, on diabetic population has revealed B12 deficiency in range of 22 percent but the study population in these studies were on average 8-9 years older than our study group, moreover methylmalonic acid levels were also measured in these studies ,thus improving sensitivity [18]. We didn't evaluate for evidence of megaloblastic anemia and concomitant folate deficiency. Therefore clinical implication of this deficiency cannot be predicted [19]. Patients with B12 deficiency were not followed to see effect of replacement of B12 [20]. Such followup would have helped to find out dose and duration of B12 replacement required in such patients.

We consider this statistically significant percentage of vitamin B12 deficiency, an important guide for physicians to always consider this as an important comorbid factor in diabetic patients particularly if they have been prescribed metformin for longer duration and in high doses [21]. Although exact clinical significance and impact of this deficiency is unknown, it is predicted that this may be a significant compounding factor towards precipitation and worsening of neuropathy and anemia in a group already predisposed to these complication due to underlying diabetes mellitus. Studies have shown that Prescription of vitamin B12 to patients of neuropathic pains may result in improvement of these symptoms [22].

## Conclusion

Our study demonstrated significantly high prevalence of vitamin B12 deficiency in patients treated with metformin with significant effect of dose and duration of metformin use on B12 levels. Physicians must recognize this Important fact and screen diabetics on metformin therapy for underlying B12 deficiency specially those presenting with neuropathic symptoms. Further studies are required to evaluate effect of B12 replacement in these patients towards reducing B12 deficiency and associated symptoms.
